# Plasma Levels of the Interleukin-1-Receptor Antagonist Are Lower in Women with Gestational Diabetes Mellitus and Are Particularly Associated with Postpartum Development of Type 2 Diabetes

**DOI:** 10.1371/journal.pone.0155701

**Published:** 2016-05-25

**Authors:** Pernilla Katra, Jonatan Dereke, Charlotta Nilsson, Magnus Hillman

**Affiliations:** 1 Department of Clinical Sciences, Diabetes Research Laboratory, Lund University, Lund, Sweden; 2 Department of Pediatrics, Helsingborg Hospital, Helsingborg, Sweden; Virgen Macarena University Hospital, School of Medicine, University of Seville, SPAIN

## Abstract

Diabetes mellitus is a group of diseases characterized by chronic hyperglycemia. Women who develops hyperglycemia for the first time during pregnancy receive the diagnosis gestational diabetes mellitus (GDM). Presently, there is no consensus about the diagnostic criteria for GDM. A majority of these women subsequently develop postpartum overt diabetes making it important to identify these patients as early as possible. In this study we investigated if plasma levels of the interleukin-1 receptor antagonist (IL-1Ra), an endogenous inhibitor of IL-1 signaling, can be used as a complementary biomarker for diagnosing GDM and predicting postpartum development of overt diabetes mellitus. Patients participating in this study (n = 227) were diagnosed with their first GDM 2004–2013 at Lund University Hospital, Lund, Sweden. Healthy pregnant volunteers (n = 156) were recruited from women’s welfare centers in the same region 2014–2015. Levels of IL-1Ra and C-peptide were analyzed in ethylenediaminetetraacetic acid (EDTA)-plasma or serum using enzyme linked immunosorbent assay (ELISA). GDM patients had significantly lower levels of IL-1Ra than the control group (p = 0.012). In addition, GDM patients that had developed impaired glucose tolerance (IGT) or type 2 diabetes mellitus postpartum had significantly lower levels of IL-1Ra, and significantly higher levels of C-peptide than GDM patients that had not developed diabetes mellitus postpartum (p = 0.023) and (p = 0.0011) respectively. An inverse correlation was found between IL-1Ra and serum C-peptide levels in the control group (r_s_ = -0.31 p = 0.0001). Our results show that IL-1Ra might be included in a future panel of biomarkers, both for diagnosing GDM to complement blood glucose, and also identifying GDM patients that are at risk of developing type 2 diabetes mellitus postpartum. However, the ROC curve analysis provided a sensitivity of 52.2% and specificity of 67.1%, which nonetheless may not be sufficient enough to use IL-1Ra as a sole biomarker.

## Introduction

Diabetes mellitus is a group of diseases characterized by hyperglycemia due to lack of insulin or disturbances in insulin signaling. The most common forms of diabetes are type 1 and type 2 diabetes mellitus. Type 1 diabetes mellitus is an autoimmune disease that results in an insulin deficiency, whereas type 2 diabetes mellitus is characterized by peripheral insulin resistance frequently in combination with a dysfunctional insulin production. [1]

During pregnancy, the metabolic state undergoes a substantial change, which also affects insulin action and sensitivity. During the second half of pregnancy this affect is increased with resulting insulin resistance and subsequent hyperglycemia. In most cases the body is able to compensate for this with increased insulin secretion and most cases resolves with delivery. [2, 3]

Gestational diabetes mellitus (GDM) is defined by the American diabetes association as glucose intolerance first diagnosed during pregnancy. GDM affects approximately 1-14% of all pregnancies depending on the ethnicity of the patient group studied and diagnostic criteria used [1]. In southern Sweden the prevalence of GDM is 2.2% [4]. Patients with GDM are hyperglycemic and suffer from increased insulin resistance, similar to patients with type 2 diabetes mellitus [5]. Many patients with GDM develop impaired glucose tolerance (IGT) or type 2 diabetes mellitus postpartum [6]. The reported incidence of type 2 diabetes postpartum varies between 2.6–70% [7].

Measuring plasma glucose has long been the gold standard for diagnosing GDM, commonly determined with fasting plasma glucose (FPG) and oral glucose tolerance tests (OGTT). However, the diagnostic criteria for GDM varies in different countries, and there is a lack of consensus concerning the plasma glucose threshold level. The Hyperglycemia and Adverse Pregnancy Outcome (HAPO) study was designed to evaluate hyperglycemia during pregnancy in relation to the risk of adverse perinatal outcomes [8]. The HAPO study found a linear increase in risk of adverse outcomes with increasing plasma glucose, without a clear cut-off level [9]. Based on the results of the HAPO study the International Association of Diabetes and Pregnancy Study Group (IADPSG) delivered new diagnostic guidelines for GDM [10]. The new guidelines resulted in an overall prevalence of GDM of 17.8% in the HAPO patient material [11], an increase by almost 50% [12]. However, the new guidelines have been criticized by others [12, 13], and consensus is yet to be established.

Despite that GDM is acknowledged by researchers to be a complex disorder, focus for establishing diagnostic criteria has solely been on hyperglycemia, while other factors that promote the pathogenesis of the disease have received less attention. Soluble biomarkers are successfully used in the diagnosis of many diseases including type 1 diabetes mellitus [14, 15], and could together with blood glucose improve the diagnosis of GDM.

In addition, since GDM during pregnancy greatly increases the risk of postpartum development of overt diabetes mellitus [16] it is important to find biomarkers or clinical parameters that can predict postpartum development of diabetes mellitus already during pregnancy, in order to be able to provide early treatment.

The interleukin-1 (IL-1) receptor antagonist, IL-1Ra, is an endogenous IL-1 inhibitor and binds to the IL-1 receptor type 1 (IL-1RI), but it fails to induce intracellular signaling, and thus serves as a competitive inhibitor of IL-1 [17].

The recombinant IL-1Ra drug Anakinra has been shown to improve plasma glucose levels and β-cell function but not insulin resistance in patients with type 2 diabetes mellitus [18]. Subsequently it seems that IL-1Ra has positive effects on β-cell function and insulin secretion, but its effect in other tissues appears to be less beneficial. Interestingly, a knock-down study of IL-1Ra in obese insulin resistant mice showed reduced insulin resistance in the liver, and also reduced body weight and blood glucose levels [19].

The aims of this study were to investigate if levels of IL-1Ra can be used as a complementary biomarker for diagnosing GDM and predicting the development of overt diabetes mellitus postpartum.

## Materials and Methods

### Participants

First-time GDM patients (n = 227) diagnosed at Lund University Hospital, Lund, Sweden, between 2004 and 2013 were included in the study. GDM was diagnosed with a 2 hour 75g OGTT following overnight fast. The diagnostic criteria for GDM was a plasma glucose value exceeding 10 mmol/L. Some of the women had developed IGT (n = 28) or type 2 diabetes mellitus (n = 34) within 6 years (in median 3 years) after clinical onset while the majority remained normoglycemic (n = 165). A control group (n = 156) of pregnant volunteers without a family history of diabetes was recruited at women’s welfare centers in the same region (Malmö (Lindängen), Dalby and Staffanstorp) in 2014–2015. Body mass index (BMI) was available for the majority of GDM patients (n = 215) and controls (n = 147). Blood samples were drawn into ethylenediaminetetraacetic acid (EDTA)-plasma and serum tubes in the 12^th^ week of gestation from healthy controls (n = 156) and from women with a family history of diabetes mellitus or a BMI>30 (n = 139). Samples from patients without a family history of diabetes mellitus and BMI<30 were taken in the 28^th^ week of gestation (n = 88). Samples were sent to the laboratory by ordinary mail and stored at -70°C until time of analysis except for C-peptide which was analyzed immediately. This study was approved by the Regional Ethical Review Board in Lund (Regionala etikprövningsnämnden i Lund; 2014/383, 2014/744), and performed in accordance with the Declaration of Helsinki. All participants were given oral and written information about the study before giving written informed consent.

### IL-1Ra analysis

IL-1Ra was analyzed in EDTA-plasma using a commercially available enzyme linked immunosorbent assay (ELISA) kit (R&D systems, Minneapolis, MN, USA) according to the manufacturers' instructions, optimized for human plasma. Samples were diluted 1:5, or 1:20 if the concentration at dilution 1:5 was found to exceed the highest standard concentration, and analyzed in duplicates. The absorbance was measured at 450 nm and 405 nm in a FLOUstar Optima ELISA plate reader (BMG Labtech Gmbh, Ortenberg, Germany). The highest concentration in the 7-point standard dilution series was changed from 2500 pg/mL to 5000 pg/mL, since the lowest concentration was undetectable by the plate reader. The inter- and intra-coefficient of variation were 18.9% and 20.0%, respectively. Control and patient samples were alternated on each ELISA plate in order to minimize the effect of inter-variation.

### C-peptide analysis

C-peptide was analyzed in serum using a commercially available ELISA kit (Mercodia AB, Uppsala, Sweden) according to the manufacturers’ instructions. The detection limit of the assay was 25 pmol/L. The intra-assay and inter-assay coefficient of variation were 2.9–4.8% and 0.6–4.8%, respectively.

### Statistical analyses

Normal distribution was estimated using the D'Agostino-Pearson test for normality. Normally distributed data is presented as mean ± standard deviation (SD), and non-normally distributed data as median [interquartile range]. Depending on the distribution, t-test or the Mann-Whitney *U* test were performed to test for differences in mean or mean rank respectively between two groups. In order to test for differences in more than two groups, analysis of variance (ANOVA) or the Kruskal-Wallis H test was performed depending on the distribution of the parameters analyzed. The χ²-test was used to determine differences in family history of diabetes mellitus between GDM patients and controls. The Spearman rank-correlation test was performed to investigate correlations in continuous variables. The precision of the IL-1Ra ELISA as a diagnostic and prognostic tool was evaluated using a receiver operating characteristic (ROC) curve analysis. A p-value <0.05 was considered statistically significant. All statistical analyses were performed using MedCalc (MedCalc Software, Ostend, Belgium) for Windows® v12.7.0.0.

## Results

Clinical and biochemical data for controls and women with GDM and/or postpartum development of IGT or type 2 diabetes mellitus are presented in Table 1. The p-values given are calculated with ANOVA, the Kruskal-Wallis H test or χ²-test depending on the variable analyzed.

**Table 1:** Clinical and biochemical data for controls and GDM patients with and without postpartum IGT or type 2 diabetes mellitus.

	Controls (n = 156)	GDM without postpartum diabetes (n = 158)	GDM with postpartum IGT/T2DM (n = 69)	p-value
**Age (years)**	29.8 ± 5.3	32.1 ± 5.3	32.0 ± 6.0	<0.001
**Body mass index (kg/m**^**2**^**)**	25.4 [23.1–28.9]	26.3 [23.5–31.6]	28.3 [23.1–32.9]	0.053
**Family history of diabetes (yes/no)**	47/109	60/98	34/35	0.021
**C-peptide (nmol/L)**	0.47 [0.31–0.74]	0.93 [0.53–1.61]	1.30 [0.97–1.93]	<0.000001
**IL-1Ra (pg/mL)**	2902 [1074-6030]	2285 [491–5826]	883 [0–4047]	0.0033

Values are presented as mean ± SD or median [interquartile range]

### Lower levels of IL-1Ra in GDM patients

Women with GDM had significantly lower plasma levels of IL-1Ra (1964 [306–5276] pg/mL) compared to pregnant controls (2902 [1074-6030] pg/mL; p = 0.012). Performing a ROC curve analysis on plasma levels of IL-1Ra as a possible diagnostic tool for GDM generated a criterion of ≤820 pg/ml. Applying this criterion resulted in a sensitivity of 37.9% and a specificity of 79.5%.

In addition, patients with postpartum development of IGT or type 2 diabetes mellitus had significantly lower levels of IL-1Ra than GDM patients that had not developed any glucose intolerance postpartum (883 [0–4047] pg/mL and 2285 [491–5826] pg/mL respectively; p = 0.023). A ROC curve analysis of IL-1Ra as a possible prognostic tool for postpartum development yielded a criterion of ≤889pg/ml with a sensitivity and specificity of 52.2% and 67.1%, respectively. There was no statistically significant difference in IL-1Ra or C-peptide levels between samples taken from patients in the 12^th^ or 28^th^ week of gestation (p = 0.21 and p = 0.99).

### Increased levels of C-peptide in GDM patients

The controls had significantly lower levels of C-peptide (0.47 [0.31–0.74] nmol/L) than both GDM patients without postpartum glucose intolerance 0.93 [0.53–1.61] nmol/L) and GDM patients with postpartum development 1.30 [0.97–1.93] nmol/L; p<0.000001). There was also a statistically significant difference between women with and without postpartum development (p = 0.0011).

### Correlation between C-peptide and IL-1Ra

An inverse correlation between C-peptide and IL-1Ra levels was found the control group (r_s_ = -0.31, p = 0.0001), but not in the group of GDM patients (r_s_ = -0.05, p = 0.49). No significant correlation was found when subdividing patients at week 12 (rs = -0.04, p = 0.61) and at week 28 (rs = -0.06, p = 0.59). We could not observe any correlation between levels of IL-1Ra and age or BMI in any of the groups.

The data set containing raw data of IL-1Ra, C-peptide, gestational age and postpartum development is provided as Supporting Information (S1 Data set).

## Discussion

In this study we showed that GDM patients have significantly lower levels of IL-1Ra in plasma than healthy pregnant controls. This suggest that since patients with GDM have lower levels of the anti-inflammatory IL-1Ra, the biological effect of IL-1 is enhanced and thus promote the inflammatory process associated with GDM. As mentioned above, there is a need for better diagnostic markers for GDM. One possibility is to put together a panel of biomarkers used to screen for GDM. However, the ROC curve analysis of the IL-1Ra ELISA as a diagnostic tool resulted in a relatively low sensitivity and specificity. Therefore, IL-1Ra might not be a fully satisfactory biomarker on its own, but it may however play an important role in a future panel of biomarkers.

We also showed that women with GDM that developed IGT or type 2 diabetes mellitus postpartum had significantly lower levels of IL-1Ra compared to GDM patients that did not develop any postpartum glucose intolerance. The ROC curve analysis provided a sensitivity of 52.2% and specificity of 67.1%, which nonetheless may not be sufficient enough to use IL-1Ra as a sole biomarker. Therefore, we suggest that IL-1Ra could be included in a panel of biomarkers for the prediction of postpartum development of IGT or type 2 diabetes mellitus in GDM patients already during pregnancy. It is important to identify the GDM patients that will go on to develop IGT or type 2 diabetes mellitus postpartum as early as possible to be able to provide better treatment.

Levels of C-peptide were higher in patients with postpartum development of IGT or type 2 diabetes mellitus than in patients without postpartum glucose intolerance, indicative of increased insulin resistance and increased insulin secretion in the former group. In a previous study no difference in C-peptide levels was found between patients with or without postpartum development of diabetes mellitus [20]. However, that study included patients with both type 1 and type 2 diabetes mellitus.

An inverse correlation between C-peptide and IL-1Ra levels was also found, but only in the control group. Since all controls were collected in week 12 we wanted to exclude the possibility of bias due to gestational age. But no correlations were found in patients at week 12 and at week 28 which suggests that in normal pregnancy IL-1Ra promotes normal β-cell function and maintaining normal C-peptide levels. It is well known that high glucose levels are toxic for β-cells, via the induction of IL-1β. One study has reported that this glucotoxicity can be prevented by IL-1Ra thus restoring β-cell function [21]. This is well in accordance with our findings, where the controls have higher levels of IL-1Ra and also normal β-cell function as estimated with C-peptide values, whereas GDM patients have lower levels of IL-1Ra in combination with a β-cell dysfunction and generally high C-peptide values.

A strength with the study is that it is conducted in a region of Sweden where there is a screening program for GDM that includes all pregnant women performing an OGTT. Thus, all patients with GDM in the region are represented. In addition, the controls in this study are also pregnant women, which increases the reliability of our findings. Limitations of the study is that some C-peptide levels were taken fasting and some were taken non-fasting at the different women’s welfare centers. Nevertheless, this includes both GDM patients and controls. Also, the samples were taken at different gestational age, all samples from controls were taken in week 12, whereas samples from GDM patients were taken either in week 12 or 28. However, the majority of samples from the GDM patients were taken in week 12 and most importantly, there was no difference in levels of IL-1Ra or C-peptide between samples taken from patients in week 12 or 28. Furthermore, the GDM patients and control group were not matched for age. However, the difference in mean age was only 2.3 years, there are no studies that have shown that this age difference affects levels of IL-1Ra.

To our knowledge no other reports have been published concerning IL-1Ra in GDM patients, but studies have been made in patients suffering from other diseases, including metabolic syndrome and type 2 diabetes mellitus. In one of these studies IL-1Ra was analyzed in 12,885 controls and patients with metabolic syndrome or diabetes mellitus in Finland [22]. The reported values of IL-1Ra in all groups were much lower than levels found in our study even though the same ELISA kit from R&D Systems is used. However, it is not stated in the study if the samples of use were serum or plasma. It is unclear if this reflects that levels of IL-1Ra are increased in pregnancy.

In future studies plasma levels of soluble IL-1 receptor type II (IL-1RII) may be analyzed, to further investigate the role of IL-1 and inhibitors of IL-1 signaling in GDM patients. IL-1RII is a decoy receptor for IL-1, as it lacks the intracellular TIR domain that mediates signaling [23]. In addition, the correlation between IL-1Ra and C-peptide levels in the control group could be further investigated in a larger group of participants to be able to decipher the role of IL-1Ra in β-cell function.

In conclusion, this is the first study to show that GDM patients have significantly lower plasma levels of IL-1Ra than healthy pregnant women. Further, we show that GDM patients with postpartum development of IGT or type 2 diabetes mellitus have ever lower levels of IL-1Ra. In addition, we showed that GDM patients have significantly higher C-peptide levels than healthy controls and that GDM patients with postpartum development of IGT or type 2 diabetes mellitus have in turn even higher C-peptide levels. Our results show that IL-1Ra might be included in a future panel of biomarkers, both for diagnosing GDM to complement blood glucose, and also identifying GDM patients that are at risk of developing type 2 diabetes mellitus postpartum. However, the ROC curve analysis provided a sensitivity of 52.2% and specificity of 67.1%, which nonetheless may not be sufficient enough to use IL-1Ra as a sole biomarker.

## Supporting Information

S1 Data set Raw data of IL-1Ra, C-peptide, gestational age and postpartum development. (XLS)
